# Positive Microbiological Stains of Corneal Scrapings among Patients with Keratitis in a Tertiary Care Centre: A Descriptive Cross-sectional Study

**DOI:** 10.31729/jnma.7538

**Published:** 2022-06-30

**Authors:** Prija Poudyal, Sanjay Kumar Singh, Amit Rajbanshi, Afaque Anwar

**Affiliations:** 1 Department of Ophthalmic Pathology and Laboratory Medicine, Biratnagar Eye Hospital, Rani, Biratnagar, Morang, Nepal; 2 Department of Cornea, Biratnagar Eye Hospital, Rani, Biratnagar, Morang, Nepal; 3 Department of Health Education, Biratnagar Eye Hospital, Rani, Biratnagar, Morang, Nepal

**Keywords:** *keratitis*, *microscopy*, *Nepal*

## Abstract

**Introduction:**

Keratitis, an ocular emergency, requires rapid and accurate treatment to prevent vision impairment. Wet mount direct microscopy examination of corneal scraping smear using gram and 10% potassium hydroxide stain helps in early diagnosis and treatment. The main objective of this study was to find out the prevalence of positive microbiological stains of corneal scrapings among patients with keratitis in a tertiary care centre.

**Methods:**

A descriptive cross-sectional study was conducted in the Department of Ophthalmic Pathology and Laboratory Medicine from January, 2018 to December, 2019. Data collection was done after taking ethical approval from the Institutional Review Committee of the hospital (Reference number: BEH-IRC-35/A). All corneal smear samples received in this department were included in this study. Case records with incomplete data were excluded. Whole sampling was done. The data were analyzed using Statistical Package for the Social Sciences version 22.0. Frequency and percentage was calculated for binary data.

**Results:**

Among 4631 corneal scrapings, microbiological stains were positive in 3538 (76.40%) patients.

**Conclusions:**

The prevalence of positive microbiological stains of corneal scrapings in our study was higher in comparison to other studies done in similar settings. This technique could be used where culture facilities are unavailable or unaffordable.

## INTRODUCTION

Microbial keratitis causes devastating visual disability in millions of people every year worldwide and especially in Southeast Asian countries.^1^ It is an ocular emergency and requires rapid and prompt treatment to prevent visual impairment, decrease morbidity and preserve ocular integrity.^2,3^

Clinically diagnosed keratitis can have similar symptoms and signs and create confusion regarding the etiology of the infection. Simple, rapid, reliable, cost-effective direct microscopic examination using Gram and 10% potassium hydroxide (KOH) stain could provide prompt and accurate information on the causative agent to start the effective treatment and prevent morbidity.^4-6^ Culture remains the gold standard for the diagnosis of causative organisms.^7^

This study aimed to find out the prevalence of positive microbiological stains of corneal scrapings among patients with keratitis in a tertiary care center.

## METHODS

A descriptive cross-sectional study was conducted in the Department of Ophthalmic Pathology and Laboratory Medicine, Biratnagar Eye Hospital from January, 2018 to December, 2019. All corneal smear samples received in this department were included in this study. Case records with incomplete data were excluded. The ethical approval was received from the Institutional Review Committee of this hospital (Reference number: BEH-IRC-35/A). Whole sampling was done.

Two clean sterile glass slides with corneal smears along with three culture plates human blood agar, chocolate agar, and Sabouraud Dextrose Agar (SDA) inoculated with the specimens in 'C' shaped streaks were received in the department. Non-nutrient agar with *Escherichia coli* was used for *Acanthamoeba* culture if smears were positive for acanthamoeba cyst. Accompanied with these were the requests consisting of the details of the patients submitted by the Cornea Department. The Gram stain and 10% KOH mount were used to diagnose the bacterial and fungal keratitis respectively. These slides were examined under 10X and 40X for fungi and 100X oil immersion for bacteria. Blood agar and chocolate agar plates were incubated at 37° centigrade under aerobic conditions. They were evaluated at 24 and 48 hours for bacterial colonies and discarded after 72 hours if no growth was observed. Similarly, SDA plate was incubated at 25° centigrade which was observed daily in the first week and then twice weekly for fungal growth. It was discarded after two or more weeks if no growth was observed. Culture on non-nutrient agar overladen with *Escherichia coli* was incubated at 37° centigrade and observed daily for growth of *Acanthamoeba* species. It was discarded after 2 weeks if no growth was seen.

Diagnostic criteria for culture-positive bacterial keratitis were if the growth of the same organism was observed on two solid-phase media, if there was confluent growth at the site of inoculation in one solid media, if growth on one media was consistent with microscopy results, or growth of the same organism in repeated corneal scrapped material.^7^ Similarly, the diagnostic criteria for diagnosis of fungal keratitis were fungal hyphae or yeast seen on microscopic examination or growth at the site of inoculation on solid culture media.^6^

All the collected data were entered in Microsoft Excel, the analysis was done using the Statistical Package for Social Sciences (SPSS) version 22.0. Frequency and percentage was calculated for binary data.

## RESULTS

Out of 4631 corneal scraping smears of patients clinically diagnosed as infective keratitis, 3538 (76.40%) cases showed organisms using direct microscopy. The 10% KOH stain revealed 2,956 (83.55%) fungi, Gram stain showed 449 (12.69%) bacteria, and both stains revealed 127 (3.59%) mixed organisms. Out of six cases of *Acanthamoeba,* KOH showed cyst in all cases, whereas gram stain could reveal cyst in only one case (Table 1).

**Table 1:** Organisms via direct microscopy (n = 3538).

Organisms	n (%)
Fungus	2956 (83.55)
Bacteria	449 (12.69)
Mixed	127 (3.59)
*Acanthamoeba*	6 (0.17)

Culture findings revealed growth in 2,658 (57.40%) cases. Fungi were encountered the most with 2,153 (81.00%) cases detected by SDA followed by bacteria with 414 (15.58%) cases isolated by blood and chocolate agar. Eighty-seven (3.27%) cases with mixed organisms were found using all three culture media. Four (0.15%) cases of *Acanthamoeba* were positive with non-nutrient agar with *Escherichia coli.* The remaining two cases were lost for follow up so culture could not be done (Table 2).

**Table 2:** Organisms via culture (n= 2658).

Organisms	n (%)
Fungus	2153
	(81.00)
Bacteria	414 (15.58)
Mixed	87 (3.27)
*Acanthamoeba*	4 (0.15)

The culture was positive but smear was negative in 142 (3.06%) cases. Smear was positive but culture-negative in 1020 (22.0%) cases. Both smear and culture were positive in 2516 (54.3%) cases. Both smear and culture were negative in 951 (20.5%) cases (Table 3).

**Table 3:** Direct microscopy and culture (n = 4631).

Results	n (%)
Positive culture but negative smear	142 (3.06)
Positive smear but negative culture	1020 (22.02)
Positive smear and positive culture	2516 (54.33)
Negative smear and negative culture	951 (20.53)
Culture not done	2 (0.04)

Gram stain was used along with blood and chocolate agar to identify bacterial organisms in all the corneal samples. The high negative smear and culture results are because most of the organisms were fungal in nature in this study (Table 4).

**Table 4:** Gram stain with blood and chocolate culture for bacterial organisms (n = 4631).

Results	n (%)
Positive smear and positive culture	367 (7.92)
Negative smear but positive culture	134 (2.89)
Positive smear but negative culture	210 (4.53)
Negative smear and negative culture	3920 (84.65)

Similarly, 10% KOH stain and SDA medium was used for identification of fungal organisms in all corneal scrapings (Table 5).

**Table 5:** 10% KOH stain with SDA culture for fungal organisms (n = 4631).

Results	n (%)
Positive smear and positive culture	2142 (46.25)
Negative smear but positive culture	98 (2.11)
Positive smear but negative culture	946 (20.43)
Negative smear and negative culture	1445 (31.20)

## DISCUSSION

Our study revealed that direct microscopy was positive in 76.4% and culture was positive in 57.4% of total keratitis patients. The culture was positive but smear was negative in 3.06% of cases. Smear was positive but culture-negative in 22.0% of cases. In one study in Southern India, a direct microscopic examination of corneal scrapings detected microbes in 80.9% of culture-positive cases and 31.7% of culture-negative cases.^8^ A study based in Delhi, India revealed smear positive in 93.6% of cases and cultures positive in 51.46% of cases.^9^ Other studies revealed more positive cases with culture techniques compared to smear results. A study in Dharan, Eastern Nepal, reported 67.8% culture-positive cases compared to 48.0% smear-positive.^10^ Similarly, 40.0% culture-positive and 34.6% smear-positive cases were concluded in a study in Kathmandu, Nepal.^11^ Another study in Bangladesh reported cases with 81.7% culture-positive and 70.4% gram stain recovery rate.^12^ A study in Egypt had 44.5% culture positives compared to 30.4% of smear-positive cases.^13^

In our study, the high percentage of smear-positive cases may be because the scrapped material was first placed on the slides by the treating clinicians, which yielded more results especially when the material was very little. Also, the stains procedure can identify both viable and non-viable organisms whereas culture requires viable organisms to be isolated. The low culture positivity rate compared to microscopy could be because of prior topical use of antimicrobial therapy by the patients,^14^ non-availability of other differentials, selective, transport or liquid media, use of human blood agar instead of sheep blood agar, presence of non-infectious or sterile ulcers, inadequate sample and late delivery of the sample to the laboratory.

A good technique to obtain the scrapped material from the most appropriate area yields more positive results. In ulcers with a bacterial origin, the organisms are concentrated at the leading edge of the ulcer whereas, in fungal keratitis, fungal elements can be best obtained from the scrapings of the central deeper area.^15,16^ It should also be understood that in cases of previously treated patients, a false negative result could be encountered. In that case, it is advisable to discontinue the medicines for 24-48 hours and rescraping to be done.

Patient factors like poverty, non-availability of health care facilities everywhere, and inappropriate and longterm use of antimicrobials from local pharmacy shops generally lead to late presentation to eye hospitals. During this time, the size and depth of the ulcer may have added up, due to which adequate and proper samples could be obtained which led to increased positive results with the microbiological stains.^7^

The laboratory results using these stains may be varied due to differences in subjective interpretation and on the experience of the microbiologist.^15,17^ False negative smears may be because of inadequate experience to examine the proper areas in the slides, inadequate time allotment reading the slides because of a hectic schedule and also unable to distinguish between the organism and the stain debris. Some viable fungal structures can be present in the eye but cannot be grown in the laboratory due to differences in the growth environment (temperature, humidity).^15^ All samples that reveal fungal filaments in a smear may not show growth in culture. A fungal element is less likely to be misinterpreted while the culture has its own limitations.^18^ The KOH solution also plays a vital role in the diagnosis of fungal keratitis by its ability to clear the scraping of cellular debris and make the hyphal fragments more refractile on microscopic examination.^16^

The fungal organisms played a prominent role in causing infective keratitis in this hospital. 10% KOH contributed remarkably in detecting those organisms. Various studies have also established the importance of KOH smear of corneal scrapings for the diagnosis of fungal keratitis.^5,6,9^ Additionally, KOH also had greater sensitivity in diagnosing *Acanthamoeba,* as the cyst could be seen in all six cases reported in 2019 in this hospital, whereas gram stain could identify the cyst in only one case. Culture with blood agar, chocolate agar, and SDA yield no growth in all cases. In four cases, further culture was done on non-nutrient agar laden with *Escherichia coli,* which yielded growth of *Acanthamoeba* in all. The significant effects of 10% KOH in diagnosing Acanthamoeba keratitis has also been evaluated by other studies.^7,19^

Different studies have supported the fact to use of direct microscopy for rapid detection of organisms and the start of the treatment to decrease ocular morbidity and visual impairment.^7,11,18^ Corneal trauma and ulcerations are the second most common cause of unilateral blindness in Nepal.^20^ Nepal has three geographical terrains that include the mountainous, hilly, and Terai regions. Due to the difficult terrain structures, it is almost impossible for many corneal ulcer patients to access eye hospitals. Almost all of the eye hospitals in Nepal are situated in the Terai belt, Kathmandu, and Pokhara with few ones in hilly areas. Eye care centers are providing primary eye care facilities with refraction, and optical dispensing in the mountainous and hilly areas. None of the eye care centers in all three terrains have microbiology laboratory diagnostic facilities. Availability of a simple microscope at a cost of less than 1000 USD and training of the laboratory personnel to interpret the direct microscopy slides can help in the proper and early management of corneal ulcer patients in all areas. This could be highly beneficial in underdeveloped countries like ours, where regular follow-up is a hindrance, cultural facilities are not available everywhere and the price may not be affordable to most of the population.

This is a single-centre study so the findings cannot be generalized. The study is of descriptive nature so associations could not be explored.

## CONCLUSIONS

The prevalence of positive microbiological stains of corneal scrapings in our study was higher in comparison to other studies done in similar settings. Gram stain can be used in direct microscopic identification of bacterial organisms and 10% KOH in the detection of fungal filaments and cysts of Acanthamoeba. Smear microscopy played a significant role in identifying organisms in three fourth of the total cases and management of infective keratitis in less than an hour. This technique could be used where culture facilities are unavailable or unaffordable.
